# Electrochemical performance of a novel 1-(benzo[*d*]thiazol-2-yl)-3-methylguanidine as effective corrosion inhibitor for carbon steel in 1 M hydrochloric acid

**DOI:** 10.1038/s41598-025-12838-2

**Published:** 2025-08-02

**Authors:** Amal M. Abdel-karim, Rasha A. Azzam, Omnia El-Said Shehata, Mohamed A. adly, Omnia A. A. El-Shamy, Gamal A. El mahdy

**Affiliations:** 1 Physical Chemistry Department, National Research Centre, 33 El Bohouth St., Dokki, P.O.12622, Giza, Egypt; 2 Cemistry Department, Faculty of Science, Helwan University, Cairo, 11795 Egypt; 3 3Department of Analysis and Evaluation, Egyptian Petroleum Research Institute (EPRI), Nasr City, Cairo, 11727 Egypt

**Keywords:** Carbon steel, Guanidine benzothiazole, Corrosion inhibitor, PDP, EIS, Adsorption, DFT, Electrochemistry, Corrosion

## Abstract

Guanidine benzothiazole (G) and 1-(benzo[*d*]thiazol-2-yl)-3-methylguanidine (AG) were successfully synthesized and characterized by infrared (FT-IR) and nuclear magnetic resonance (NMR) spectroscopy. Both compounds act as novel corrosion inhibitors for carbon steel in 1.0 M hydrochloric acid solution. Their inhibition efficiency was evaluated using potentiodynamic polarization (PDP) and electrochemical impedance spectroscopy (EIS). Surface analysis via scanning electron microscope (SEM) and energy dispersive X-ray (EDX) confirmed the formation of a protective film on the steel surface. PDP results indicate that G and AG function as mixed-type inhibitors, with inhibition efficiency increasing with concentrations, reaching a maximum of 80% for G and 91.4% AG. The AG compound was seen to retard the rate of corrosion of carbon steel more effective than PP compound. Adsorption followed the Langmuir isotherm, suggesting monolayer adsorption on the metal surface. The high inhibition efficiencies were attributed to strong adsorption and the formation of a dense, protective barrier film. The corrosion inhibition performance of the synthesized molecule was evaluated using density functional theory (DFT). Quantum chemical parameters, including highest occupied molecular orbital (HOMO) and lowest occupied molecular orbital (LUMO), energy gap, hardness, and dipole moment, and other quantum descriptors were analyzed. Density functional theory (DTF) calculation supported the experimental finding, showing that the high efficiency correlates with a low energy gap and high dipole moment, which promote electron transfer and adsorption strength. Theoretical and experimental results showed strong agreement, confirming the potential of G and AG as effective inhibitors for carbon steel.

**Supplementary Information:**

The online version contains supplementary material available at 10.1038/s41598-025-12838-2.

## Introduction

Corrosion of carbon steel in acidic environments is a significant challenging that attracts attention from both academia and industry. Acidic solutions take more attention due to their extensive application in industrial cleaning and pickling processes for various metal equipment^1,2^. The acid pickling of carbon steel is a vital step for removing oxides, making it is essential to introduce inhibitors during this process to minimize metal dissolution and reduce acid consumption^3^.

Organic synthesis allows scientists to create and modify organic substances through a variety of reactions and procedures that have been and continue to be rapidly and continuously evolving. As a result, new or improved processes for manufacturing a wide range of very significant compounds in various sectors are published on a daily basis^4^.

Organic molecules offer a wide range of applications, including the development of novel organic compounds as effective corrosion inhibitors. It is considered as one of the most practical and widely used methods of protecting metals from corrosion. The core of such compounds is the heterocyclic ring, which contains heteroatoms with high electron densities such as oxygen, phosphours, nitrogen, sulfur or other groups with multiple bonds. These heteroatoms serve as adsorption sites, playing a principle role in corrosion inhibition. The inhibitory effect of these organic compounds is mainly attributed to adsorption (physisorption or chemisorptions), driven by the interaction between the polar centers of the inhibitor and the active sites on the metal surface^5^.

Physisorption arises from electrostatic attractive forces between organic ions or dipoles of the inhibitor and the electrically charged metal surface. In contrast, chemisorption occurs due to interaction of unshared electron pairs or π electrons in the adsorbate with the metal, forming a coordinate bond. This process is facilitated by the presence of hetroatoms with lone-pair electrons and/or aromatic rings in the inhibitor molecules^6,7^. Through this mechanism, the adsorption of the inhibitor onto active sites on the metal surface reduces either the anodic or cathodic reaction, or both, thereby mitigating corrosion. The extent of adsorption, and consequently the effectiveness of the inhibitor, is influenced by several factors, including the electronic characteristic of the adsorbate, the chemical composition of the solution, the nature of the metal surface, and the electrochemical potential at the metal-solution interface.

Among the organic compounds which are effectiveas inhibitors in acid solutions there are nitrogen containing compounds^8^ such as pyridazine^9^, pyridine, quinoline^10^, pyrazine^11^, pyrazole^12^, acridine^13^, benzimidazole^14^,, and triazole^15^ have a nitrogen heteroatom, mercapto functional compounds^16^.

Benzothaizole derivatives exhibit remarkable biological activities^17,18^ and are known for their effectiveness as corrosion inhibitors due to it has three adsorption sites: the nitrogen atom with its lone pair of electrons, the sulphur atom, and the aromatic rings. One of benzothiazole derivatives which show high inhibitor efficiency as corrosion inhibitors with concentration is 2- hydrazinobenzothiazole (HBT). Developing cost effective, environmentally friendly, and efficient corrosion inhibitors with structural similarities to HBT has gained attention, with new derivatives like guanidine benzothiazole (GBC) being a focus of particular interest^19^.

According to a survey of the literature, highlight the application of benzothiazoles as effective corrosion inhibitors for steel and copper. Their planar fused heterocyclic molecular structure enables strong interactions with metal ions through donor atoms such as S and N forming of stable metal complexes. The benzothiazole molecule features adsorption-friendly sites, including the nitrogen atom with its lone pair of electrons, the sulphur atom, and the aromatic rings, making it highly effective for surface adsorption.

For example, 3-(piperazin-1-yl) benzo(*d*) isothiazole (PBIT) has been identified as an excellent corrosion inhibitor for steel in mild hydrochloric acid solutions. Studies have shown that its inhibition efficiency increases proportionally with the inhibitor concentration, while the corrosion rate decreases. Similarly, 2-mercaptobenzothiazole (MBT) and 2-aminobenzothiazole (ABT) have demonstrated significant corrosion inhibition effects on carbon steel immersed in 1 M hydrochloric acid solution^20,21^.

The use of computational chemistry to study the corrosion protection capabilities of organic compounds has gained significant attention in recent years. Numerous academics declare that the quantum-chemical methods are among the most valuable tools for evaluating the performance of metal corrosion inhibitors. Additionally, theoretical approaches are a practical means to ascertain the inhibitor-metal interactions because they do not necessitate time-consuming experimental investigations^22^.

To the best of our knowledge, guanidine benzothiazole (G) and 1-(benzo[d]thiazol-2-yl)−3-methylguanidine (AG**)** have not been previously reported or examined as corrosion inhibitors for carbon steel in hydrochloric acid solution. This study aims to synthesize and characterize new derivatives of guanidine benzothiazole using FTIR, and1H, 13 C NMR spectroscopy, and evaluate their effectiveness as corrosion inhibitors for carbon steel in hydrochloric acid. Their performance was assessed using electrochemical techniques, including potentiodynamic polarization and electrochemical impedance spectroscopy. The selection of these compounds is based on their molecular structure, characterized by a high content of heteroatoms (N and S) with lone pairs of electrons and aromatic ring with delocalized π electrons, making them excellent candidates for adsorption onto metal surface. Additionally, surface morphology analysis using scanning electron microscopy (SEM) and energy dispersive X-ray (EDX) confirmed the effectiveness of the inhibitors. The adsorption behavior was further studied to identify the suitable adsorption isotherm and calculate the adsorption energy.

## Experimental

### Synthesis and characterization

The reagents and solvents used in this study were obtained in commercially available grade purity, *o*-aminothiophenol 98% (Alfa Aesar), cyanoguanidine 99%(Alfa Aesar), and methyl iodide stabilized (Merck). Guanidine benzothiazole (G) was synthesized following a previously reported procedure^23^. The derivative 1-(benzo[*d*]thiazol-2-yl)−3-methylguanidine (AG) was prepared by the reacting 0.002 mol of methyl iodide (0.124mL) with 0.002 mol of benzothiazol guanidine (0.4 g) in 10 mL of ethyl alcohol. The mixture was stirred under reflux for 3 h. After cooling, the alcohol was evaporated and the resulting solid product was washed and crystallized using ethanol. The progress of the reaction was monitored by thin-layer chromatography (TLC) on aluminum sheets coated with silica gel MERCK 60 F 254, and visualization was performed using UV lamp. Melting points were determined using an SMP3 melting point apparatus with open capillary tubes, and the values are uncorrected.

Elemental analyses were performed to confirm the composition of the synthesized compounds. IR spectra were recorded on FTIR plus 460 or Pye Unicam SP-1000 spectrophotometer with KBr pellets. ^1^H NMR (400 MHz) and ^13^C NMR (100 MHz) spectra were done in NMR unit at Faculty of Pharmacy, Beni-Swef University. These spectra were recorded using a Bruker advance (III) Ultra Shield NMR spectrometer with DMSO-*d*_6_ as the solvent and tetramethylsilane (TMS) as an internal standard. Chemical shifts are reported as δ_ppm_ units. Supplementary S1 and S2 show IR spectra of G and AG, respectively. Supplementary S3 and S4 show 1H NMR spectroscopies of two compounds. 13 C NMR spectroscopies presented in Supplementary S5.

### Electrochemical measurements

Carbon steel (CS) specimens (composition: 2.68% C, 0.36% Si, 0.24% Cr, and 96.72% Fe), with dimensions of 1 × 2 × 0.5 cm were used for electrochemical measurements. Prior to testing, specimens were polished with emery papers of various grades, washed with acetone, rinsed with double distilled water, and dried. The electrode was then immediately immersed in the test solution without or with the desired concentration of the investigated inhibitors. The test solutions contained analytical -grade 37% hydrochloric acid (Merck).

The guanidine benzothiazole (G) and 1-benzothiazol-2 yl −3- methylguanidine (AG) concentrations of 125, 250, and 500 ppm were chosen based on initial screening tests to determine effective levels that produce clear inhibition effect. This range allows for evaluating how inhibitor performance varies with concentration, ensuring sufficient adsorption on the metal surface. Additionally, these concentration levels align with those reported in prior research on similar organic inhibitors, enabling reliable comparison and interpretation of the results^19^.The synthesized inhibitors G and AG were completely soluble and stably dispersed in 1.0 M HCl at the tested concentrations, with no observable precipitation during the preparation or testing procedures.

Electrochemical measurements were conducted using Autolab Potentiostat/Galvanostat PGSTAT 302 N with electrochemical cell with three electrode Pyrex glass cell, a reference electrode (silver/silver chloride), the counter electrode (platinum), and working electrode (CS with subjected 1cm^2^ area). The working electrode was first immersed in the test solution at room temperature for establishing a steady state open circuit potential prior to the electrochemical measurements. Electrochemical tests were conducted three times to verify reproducibility. The results presented are the averages of these independent trials, with standard deviations falling within acceptable limits. Data were chosen based on stable and consistent measurements under carefully controlled conditions, such as fixed temperature, solution concentration, and surface of the electrode.

#### Electrochemical impedance spectroscopy (EIS)

AC impedance responses were recorded at open circuit potential (OCP) with a sinusoidal excitation signal 10.0 mV peak-to-peak in the frequency range from 0.01 Hz to 100 kHz. Impedance (Z) and phase shift (θ) were included. Data obtained were analyzed with software Nova 1.11.2, https://www.metrohm.Autolab.com. Three sets were repeated for tested electrolyte to achieve accurate results at room temperature.

1 $$\:IE\%=\frac{{R}_{i}-{R}_{o}}{{R}_{i}}x100\:$$

Where *R*_o_ and *R*_i_ are the resistance without and with G and EG inhibitors, respectively.

#### Potentiodynamic polarization (PDP)

The potentiodynamic polarization curves were recorded after reached open circuit potential (OCP) to study the corrosion efficiency of prepared inhibitors G and EG at various concentrations, all polarization measurements were performed in the potential of −1000 to −200 mv, with scan rates of 1 mVs^−1^for different G and EG concentrations.

Tafel parameters were obtained by extrapolating the linear regions of the cathodic and anodic polarization curves where a clear logarithmic relationship between current density and potential was observed; using automated fitting tools provided by the electrochemical analysis software. The inhibition efficiency (IE %) has been calculated using Tafel extrapolation method by

2 $$\:IE\%=\frac{{i}_{o}-{i}_{i}}{{i}_{0}}x100$$

Where, *i*_o_, *i*_i_ of the current densities without and with G and EG inhibitors, respectively.

### Surface morphology

Scanning electron microscopy (SEM) was used to examine the surface morphology of carbon steel samples in the presence and absence of G and AG inhibitors. A focused electron beam was employed to scan the corroded area, and the generated signals provided detailed information about the surface topography. SEM analysis was conducted on carbon steel specimens immersed in 1.0 M HCl without and with inhibitors 500 ppm G and AG inhibitors using a JSM-6510 microscope equipped with an energy dispersive X-ray spectrometer (Quantax75).

### Theoretical calculation

Density Functional Theory (DFT) calculations were performed to investigate the electronic properties and reactivity of both neutral and protonated forms of the inhibitors (G and AG). All calculations were carried out using the Hyper Chem 8.0.10 software, chemistry software, hyperchem, molecular modeling http://www.hypercubeusa.com. Geometry optimization was performed in the gas phase using the B3LYP functional with the 6-31G(d, p) basis set. No symmetry constraints were applied during the optimization. Quantum chemical descriptors were derived based on the energies of the highest and lowest occupied molecular orbital *E*_HOMO_ and E_LUMO_, respectively^24^. Dipole moment (D) and different quantum descriptors were calculated Such as the energy gap ΔE_g_ (*E*_LUMO_ – *E*_HOMO_), Mullikencharge population, Ionization potential (*I*=-*E*_HOMO_), electron affinity (*A*= -*E*_LUMO_), absolute electronegativity, *X*, (average of ionization potential and electron affinity), and global hardness *(η*= (I-*A*)/2, softness (Ϭ),electrophilicity index (ω), and Mulliken atomic charges were calculated^25^.These parameters provide insights into the electronic properties and reactivity of the inhibitors. In addition, the adsorption behavior and active sites of the inhibitors were predicted based on the distribution of Mulliken charges and the energy values, helping to correlate experimental inhibition efficiency with theoretical predictions.

## Results and discussion

### Synthesis and characterization

**Scheme 1:**
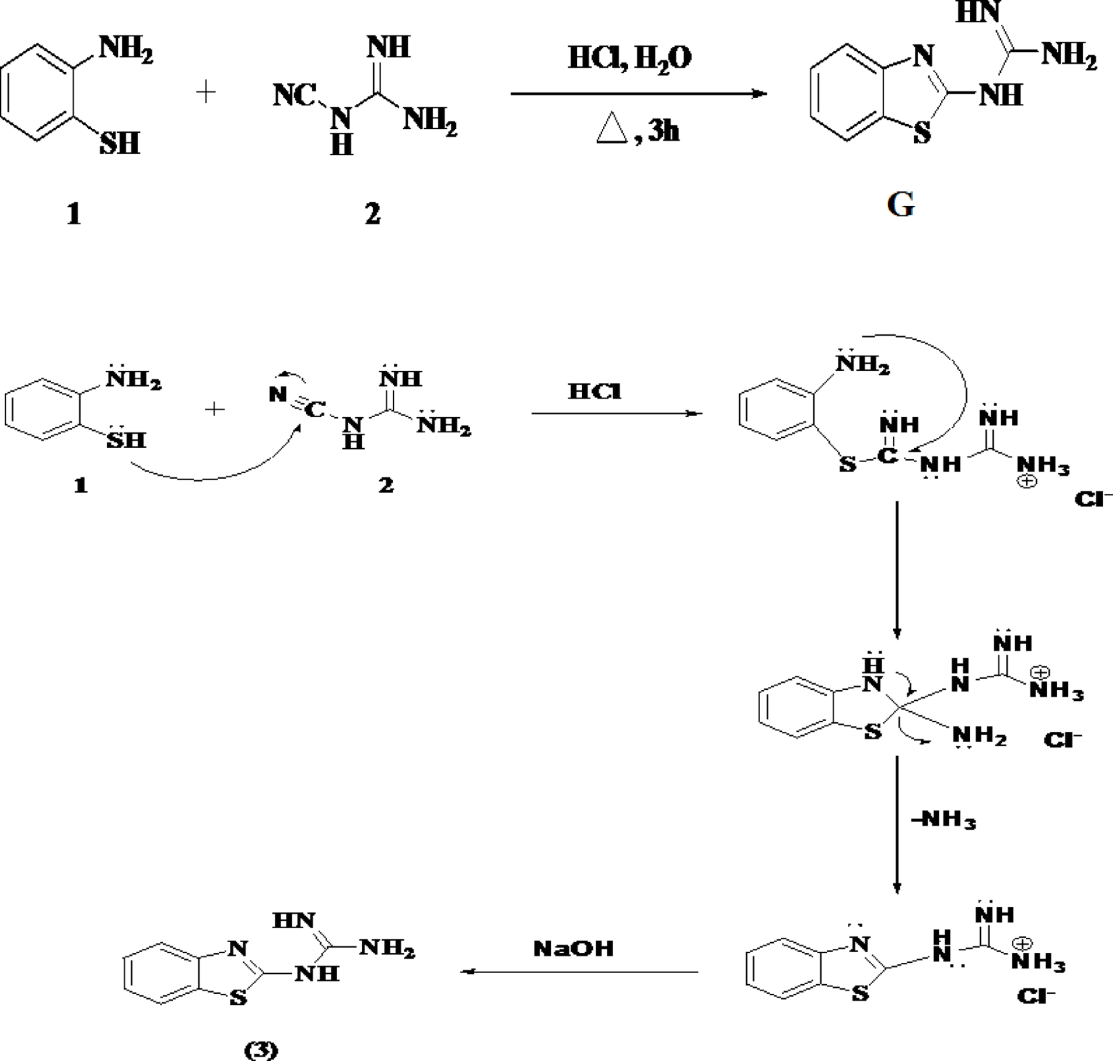
Synthesis of 2-benzothiazolylguanidine **(G)**^23^.

Several methods are available for synthesizing the benzolthiazole ring, with the most common involving the reaction of *o*-aminothiophenol with nitrile derivatives. To obtain 2-benzothiazolylguanidine **(G)** in a high yield, *o*-aminothiophenol**(1)** was reacted with 1-cyanoguanidine **(2)** via a cyclocondensation reaction in an acidic medium, as illustrated in Scheme 1^23^.

Grayish while powder; yield 90%;mp;170–171 °C, IR(KBr), υ 3350CM-1(N-H),1620(C = N),1450(C = = C),1270(C-N)740(C-S) CM-1 ^1^H NMR (300 MHz, DMSO-*d*_6_):δ5.54 (br, 2 H, NH_2_), 7.10–7.14 (m, ^1^H, benzothiazole-H), 7.25–7.29 (m, ^1^H, benzothiazole-H), 7.56 (d, *J* = 8.2 Hz, ^1^H, benzothiazole-H), 7.73 (d, *J* = 8.2 Hz, ^1^H, benzothiazole-H), 8.74 (s, 2 H, NH). ^13^C NMR (100 MHz, DMSO-*d*_6_): 119.5, 120.3, 122.6, 125.1, 135.2, 150.9, 164.6, 174.0 (Ar-C).

### 1-(benzo[*d*]thiazol-2-yl)−3-methylguanidine (AG)

**Scheme 2:**
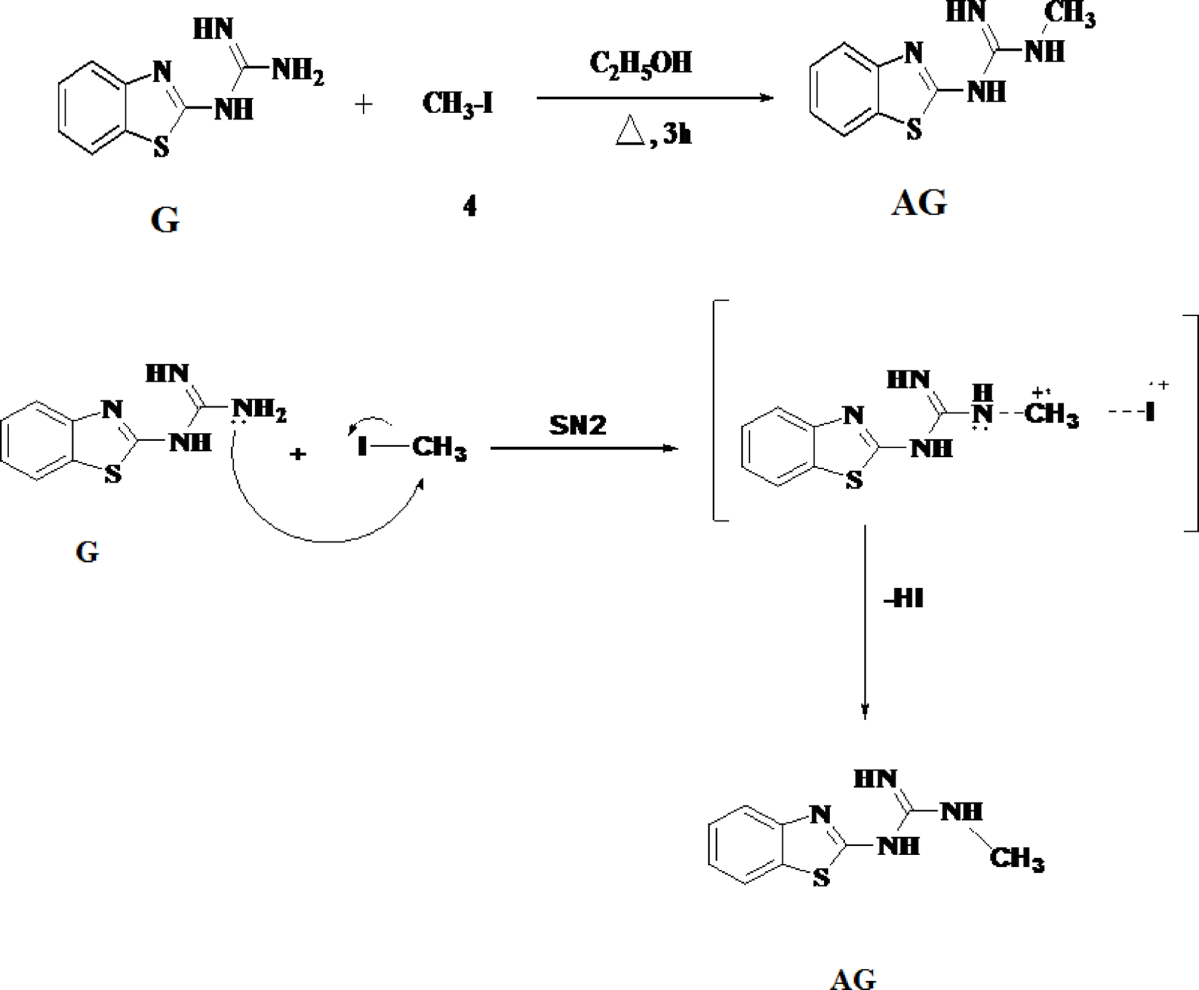
Synthesis of 1-(benzo[*d*]thiazol-2-yl)−3-methylguanidine **(AG)**.

2-Benzothiazolyl guanidine (G) served as the starting material for synthesis of various benzothiazole derivatives. A new benzothiazole derivative was symthesized by the reacting methyl iodide (4) with 2-benzothiazolylguanidine (G), as depicted in Scheme 2.

White powder; yield 80%; mp;176–177 °C, IR (KBr, cm^−1^): υ 3251 (NH), 3073 (ArCH), 2752 (Aliph-H), 1610 (C = C),1531 (C = N); ^1^H NMR (300 MHz, DMSO-*d*_6_): δ3.19 (s, 3H, CH_3_), 7.43 (t, *J* = 8.0 Hz, ^1^H, benzothiazole-H), 7.54(t, *J* = 8.0 Hz, ^1^H, benzothiazole-H), 7.89 (d, *J* = 8.0 Hz, ^1^H, benzothiazole-H), 8.12 (d, *J* = 8.0 Hz, ^1^H, benzothiazole-H), 8.69 (s, 3H, ^3^NH). ^13^C NMR (100 MHz, DMSO-*d*_6_): 39.7 (CH_3_), 122.1, 122.5, 125.5, 127.5, 131.8, 149.7, 156.6, 164.3.

The reaction proceeded via a nucleophilic substitution mechanism, where the amino group in 2-benzothiazolylguanidine (G) replaced the iodine atom in methyl iodide. The structure of the resultant product (AG) was confirmed through IR and NMR spectroscopy, with the spectra data compared to that of the starting chemical, 2-benzothiazolylguanidine (G).

The presence of the benzothiazole ring in both compounds (G) and (AG) was confirmed by ^1^H NMR spectra, which showed two triplet and two doublet peaks at δ7.30–8.20 ppm. An additional single peak at δ3.19 ppm was observed, which represents the methyl group in compound (AG). Furthermore, the NH_2_ peak at δ5.54 ppm in compound (3) disappeared in compound (AG), indicating that the methyl group had bonded to the amino group. In ^13^C NMR spectra of compound (AG), the carbon of methyl group was represented by peak atδ39.7 ppm, further confirming the structure of the derivative. The confirmed structure, IUPAC name and the chemical formula for **G** and **AG** are shown in Table 1.

**Table 1:**
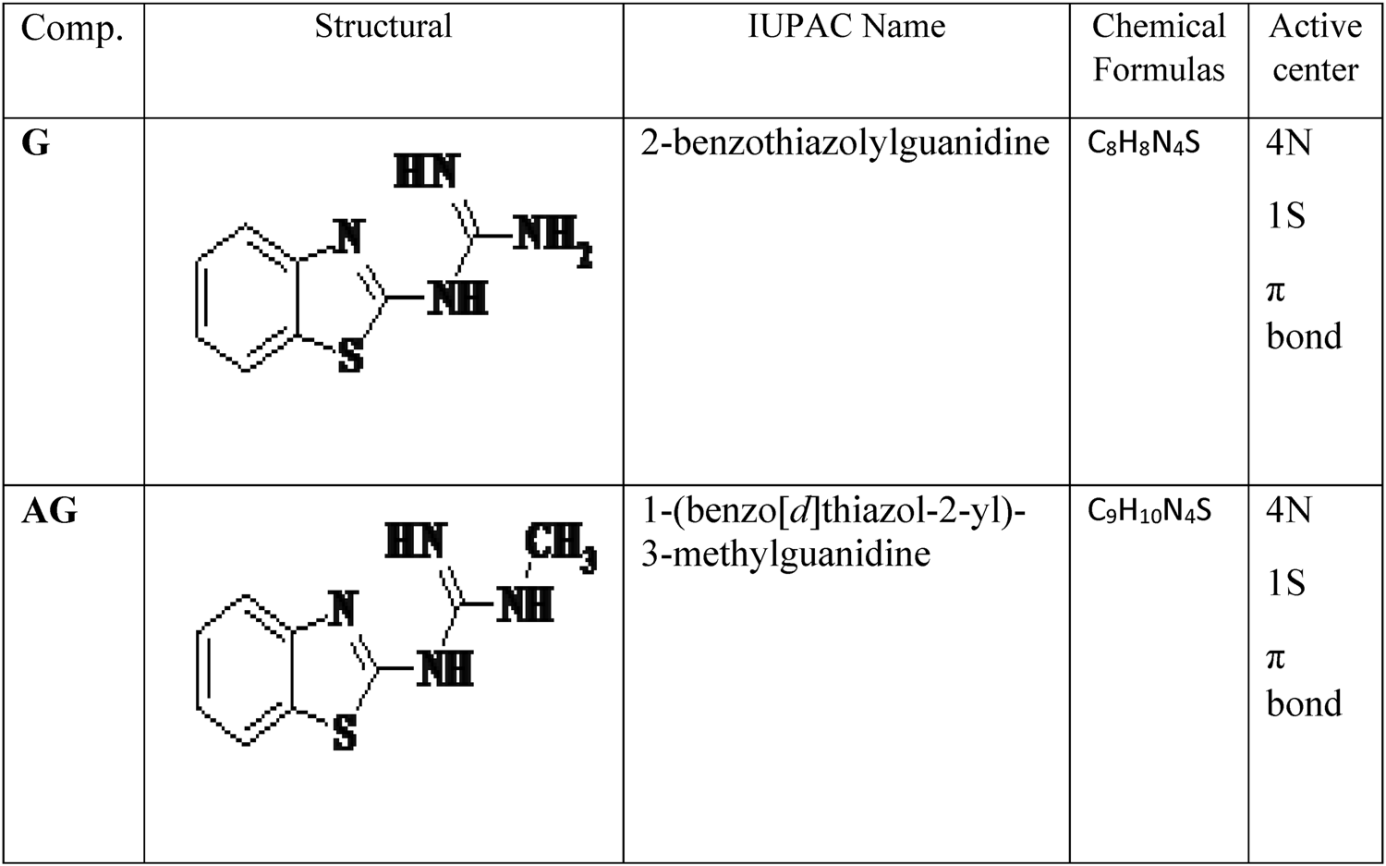
The structural, IUPAC name, chemical formula of G and AG.

### **Electrochemical impedance spectroscopy measurements** (**EIS)**

**Fig. 1:**
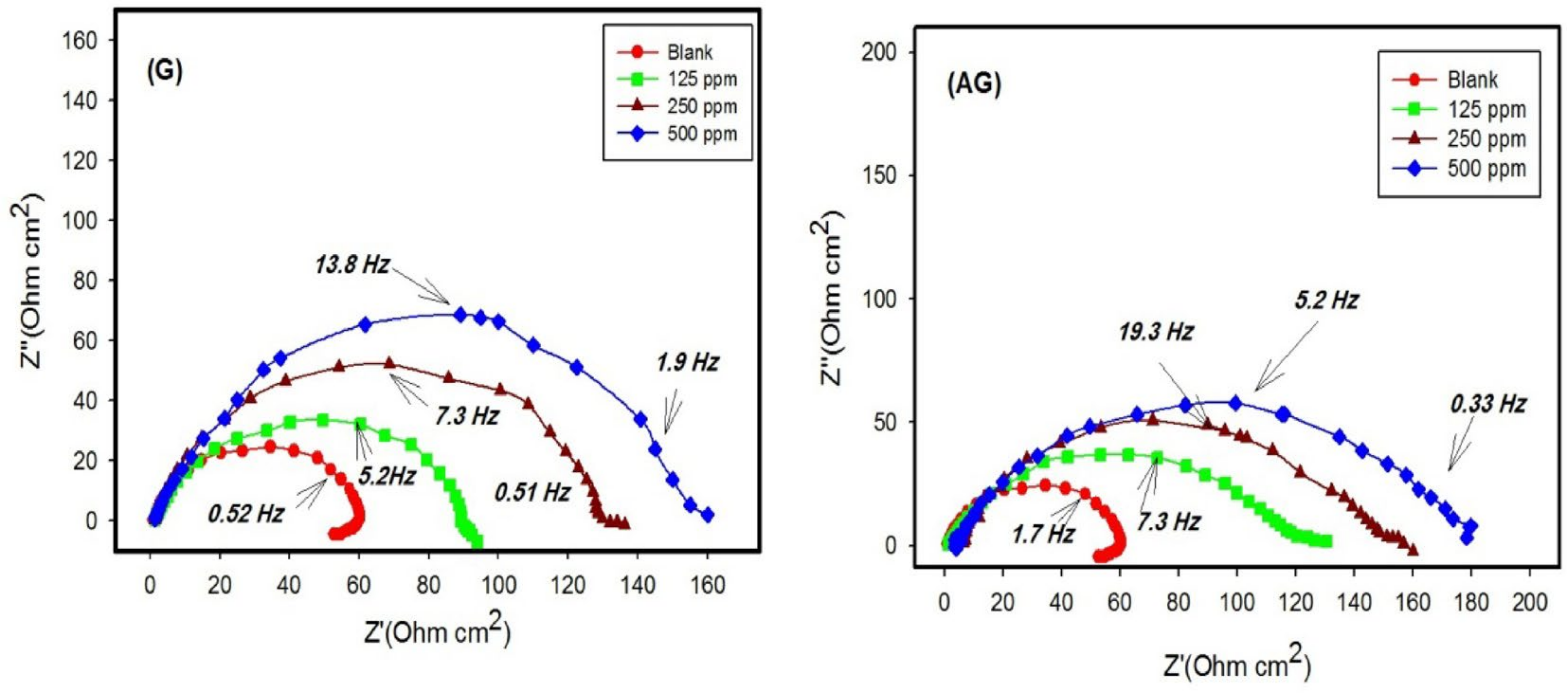
Nyquist plots for carbon steel in 1.0 M HCl with and without various concentrations of inhibitors G and AG at ambient temperature.

**Fig. 2:**
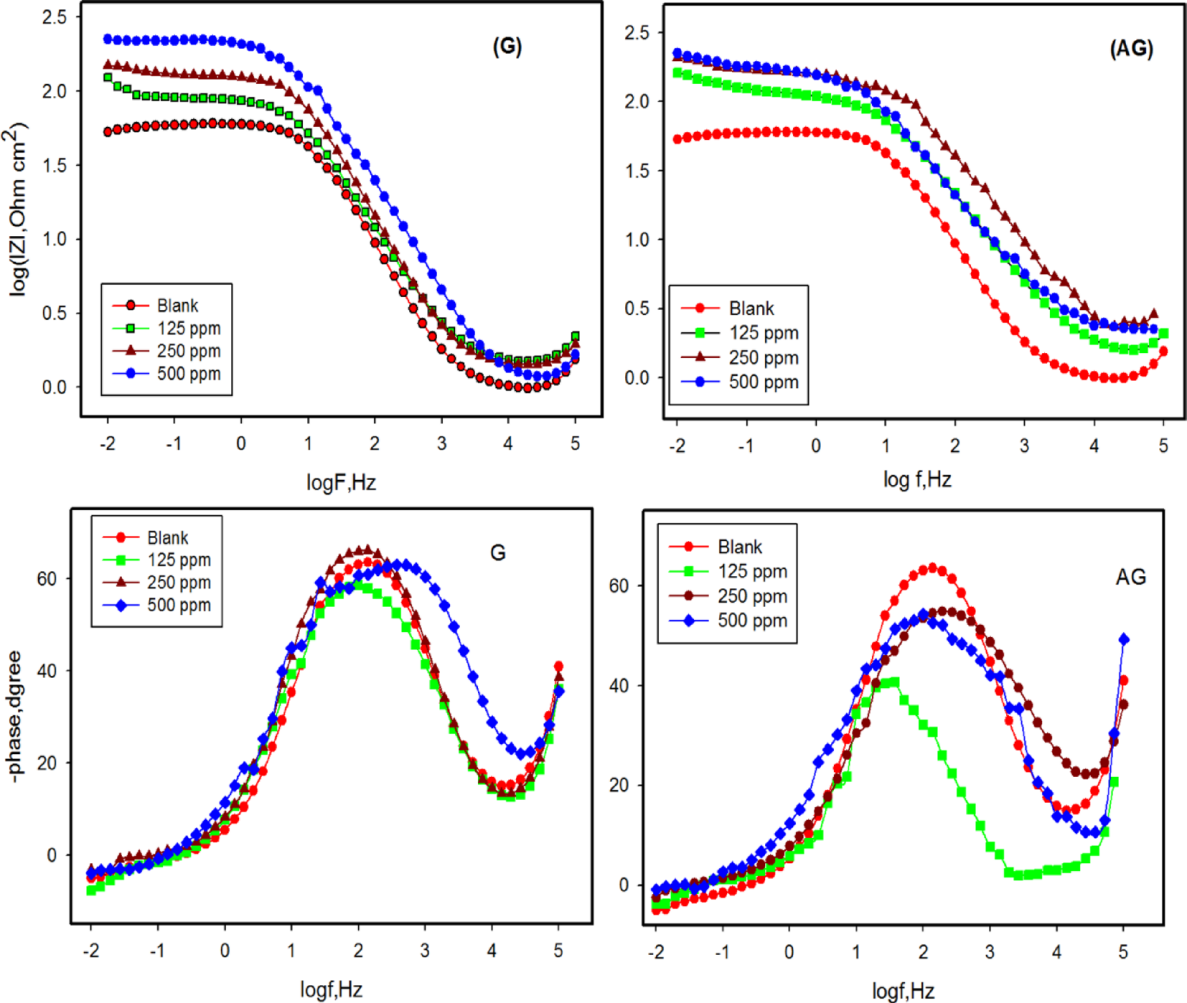
Bode and phase plots for carbon steel in 1.0 M HCl with and without various concentrations of inhibitors G and AG at ambient temperature.

Impedance experiments provided valuable insights into the characteristics and kinetics of electrochemical processes occurring at the metal/acid interface and the modifications induced by the tested inhibitors. The impedance responses are presented as Nyquist plots in Fig. 1 and Bode/phase plots Fig. 2.

The Nyquist diagrams (Fig. 1) illustrate the impedance behavior of carbon steel in 1.0 M HCl containing various concentrations of inhibitors (G and AG). The impedance exhibited a single capacitive loop, corresponding to a single time constant, with its magnitude increasing as the inhibitor concentration rose. The capacitive loop is associated with a faradic process involving a charge transfer resistance in parallel with a double-layer capacitance element^6^. This behavior suggests that as the concentration of the inhibitors increased, more inhibitor molecules adsorbed onto the CS surface. As a result, both G and AG enhanced the charge transfer resistance at the electrode-electrolyte interface. This indicates that the corrosion inhibition of CS in 1.0 M HCl mainly involves a charge transfer process. The generation of microscopic roughness on the surface during the corrosion contributes to this behavior. Both uninhibited and inhibited solutions exhibit a single capacitive loop, as reflected in the Bode plots (Fig. 2), which corresponds to the charge transfer process of corrosion. The bode-phase plots show a single peak, confirming that only one process occurs on the metal surface. Additionally, the phase angle shifts to lower frequencies in the presence of inhibitor compared to that of blank solution, suggesting an increased adsorption of inhibitor molecules on the metal surface and enhanced capacitive behavior^26^.

**Fig. 3:**
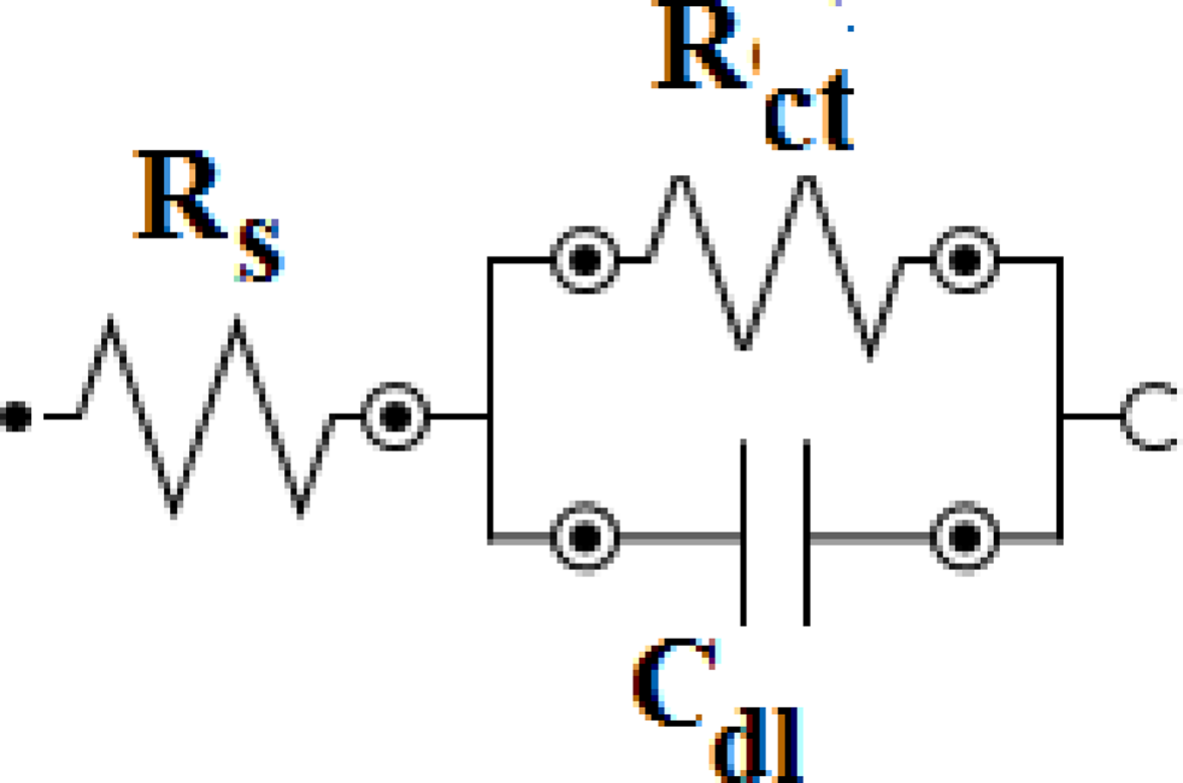
Equivalent circuit models used to simulate the impedance behavior of carbon steel in tested conditions.

The corresponding electrical equivalent circuit model is shown in Fig. 3^27^. The parameters derived from electrochemical impedance spectroscopy (EIS) analysis, including the *R*_s_ is the solution resistance, double layer capacitance (*C*_dl_), charge transfer resistance (*R*_ct_) and the inhibition efficiency (*E*_EIS_ %) are summarized in Table 2.

The inhibition efficiency was calculated to compare the performance of both techniques.

The values of R_s_ remained relatively stable, confirming consistent solution conductivity.

These results indicate that *R*_ct_ increased while *C*_dl_ deceased as the inhibitor concentration increased. The rise in *R*_ct_ can be attributed to the formation of a protective film on the carbon steel/solution interface that reduces charge transfer across the metal/solution interface. These trends support the adsorption of the inhibitor molecules and the development of a compact film that hinders corrosive species from reaching the metal surface^28^. The reduction in *C*_dl_ is expected due to a decrease in the local dielectric constant or an increase in the thickness of the electrical double layer, both of which result from the adsorption of inhibitor molecules onto the surface. Functional groups in the inhibitors (–NH₃, S, NH, CH₃) reduce water molecule mobility and the dielectric constant, supporting both physisorption and chemisorption mechanisms. Table 2 shows that inhibitors G and AG enhance adsorption at the metal/solution interface by replacing water molecules, thereby reducing metal dissolution^29^.

The adequacy of the proposed equivalent circuit was confirmed by the chi-square (χ²) values presented in Table 2. Lower χ² values reflect a stronger correlation between the fitted and experimental data, indicating that the circuit model accurately represents the system’s electrochemical behavior.

**Table 2:** Electrochemical impedance parameters and corresponding Inhibition efficiencies for carbon steel in 1.0 M HCl in the presence and absence of various concentrations of G and AG at room temperature.

Inhibitors	Conc. (ppm)	*R*_s_ (Ω)	*R*_ct_ (Ω)	C_dl_ (µFcm^−2^)	E%	d nm	^χ2^x10^−4^
Blank	0	1.62	40	179	----	5.50 × 10^−3^	0.74
G	125	1.65	82	147	51.1	6.80 × 10^−3^	0.60
	250	1.50	122	118	67.2	8.40 × 10^−3^	0.67
	500	4.40	151	66.6	73.5	1.36 × 10^−2^	0.50
AG	125	1.77	101	103	60.4	9.70 × 10^−3^	0.40
	250	5.20	135	72.4	70.4	1.38 × 10^−2^	0.38
	500	1.57	246	54.6	84.0	1.83 × 10^−2^	0.34

The thickness of the oxide film (d) is inversely proportional to its capacitance and can calculated using the relation: *d* = ε_o_ε_r_ A/C, where ε_o_ is the permittivity of the space (εo = 8.85 × 10^−14^ F/cm), ε_r_ is the relative dielectric constant of the oxide film and A represent the sample area (cm^2^)^30^.

In 1.0 M HCl solution, the barrier layer thickness for bare carbon steel is the thinnest but increases proportionally with the concentration of inhibitors. Additionally, the inhibition efficiencies in 1.0 M HCl with 500 ppm of G and AG are 73.5% and 84.0%, respectively, confirming AG as effective inhibitors for carbon steel in 1.0 M HCl solution.

### Potentiodynamic polarization curves (PDP)

**Fig. 4:**
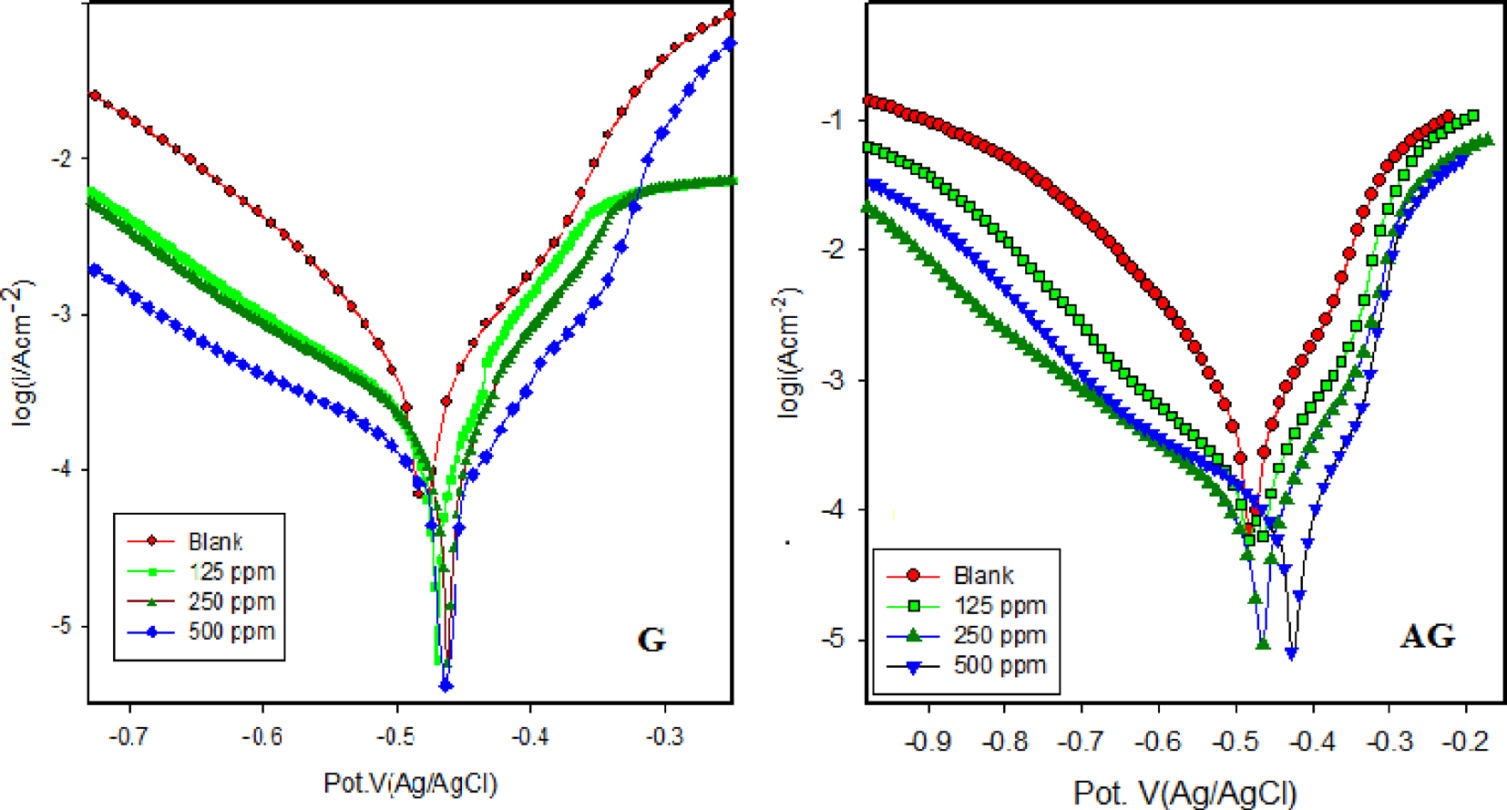
Potentiodynamic polarization plots for carbon steel in 1.0 M HCl with and without various concentrations of inhibitors G and AG at ambient temperature.

The potentiodynamic polarization curves for carbon steel in 1.0 M HCl, with and without varying concentrations of G and AG, are presented in Fig. 4. Tafel plots reveal that both the anodic and cathodic branches are affected by the addition of the synthesized inhibitors. The anodic branch represents the iron dissolution process (oxidation) (Fe------ Fe^2+^ + 2e), while the cathodic branch corresponding to hydrogen evolution (reduction) (2 H^+^+2e-----H_2_).

The inhibition efficiency improves with increasing concentrations of G and AG due to their adsorption onto the steel surface^31^. The adsorption occurs via electron pair of heteroatoms (S and N), and the π electron of benzene rings in the inhibitors’ molecular structure, blocking the steel surface and mitigating corrosion in HCl media.

Corrosion parameters, including corrosion potential E_corr_ and corrosion current density I_corr_, were performed from the intersection of the linear anodic and cathodic branches. Additionally, the cathodic Tafel slope *β*_c_ and the anodic Tafel slope *β*_a_ were calculated using the Tafel extrapolation method with software for i/E analysis of the PDP curves, as summarized in Table 3. The deviation of *β* values from those in the uninhibited solution confirms that the inhibitors interfere with both anodic and cathodic reaction kinetics and the inhibition mechanism involve surface coverage effects.

The change observed in the slope of the cathodic and anodic current- potential lines indicate alterations in the reaction mechanisms for both anodic and cathodic processes in the inhibited solution over time. The corrosion current density significantly decreases in the presence of inhibitors compared to the blank solution, demonstrating the inhibitors’ protective nature. Specifically, the current density is 466.1 uA cm^−^² in the blank solution reduced to 93.03uA cm^−^² and 40.08uA cm^−^² at 500 ppm concentrations of G and AG, respectively.

Additionally, the corrosion potentials of carbon steel in 0.1 M HCl with G and AG shift to more positive value. This shift, being less than ± 85 mV relative to the blank the corrosion potential, indicates that the both inhibitors act as mixed-type inhibitors with predominant anodic influence^32^.

AG and G act as adsorptive inhibitors, reducing anodic dissolution while also hindering the hydrogen evolution reaction by blocking active reaction sites on the CS surface. The polarization resistance (*R*_p_) of carbon steel significantly increases from 58.58 Ohm in the blank solution to 281.5 and 457.3 at 500 ppm concentrations of G and AG, respectively, further confirming their effectiveness in corrosion inhibition.

The corrosion inhibition efficiency (*E*_PDP_ %) can be calculated from the Icorr values and the degree of surface coverage (θ) using *θ* = *E*/100^29^.

**Fig. 5:**
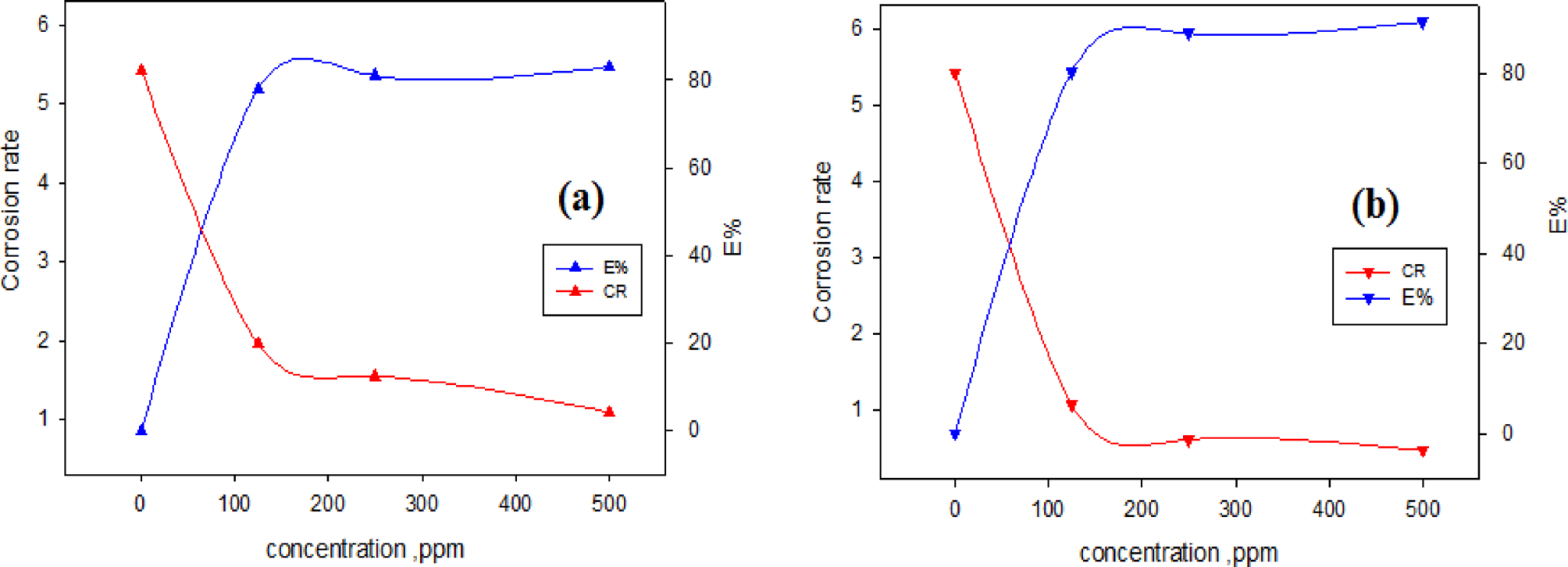
The variation of corrosion rate (CR) and inhibition efficiency (E %) with various concentration of inhibitors (a) G and (b) AG in 1.0 M HCl at room temperature.

The corrosion rate and inhibition efficiency (E_PDP_ %) as functions of inhibitor concentration are illustrated in Fig. 5 (a, b). The data demonstrate a decrease in corrosion rate and an increase in inhibition efficiency with rising concentrations of G and AG.

while the increases. Across all tested solution, the corrosion rate is especially lower with AG than with G, indicating superior corrosion resistance and a more stable protective film.

**Table 3:** The polarization parameters for carbon steel with and without various concentrations of inhibitors in 1.0 M HCl at ambient temperature.

Inhibitors	Conc ppm	βa mV/dec	-Βc mV/dec	- E_corr_ mV	i_corr_ uA/cm²	CR mm/y	*R*_pol_ (Ω)	E%	θ	SD
Blank	0	122.4	129.2	−479	466.1	5.42	58.58	----	----	---
G	125	93.0	184.7	−463	167.58	1.95	160.4	64.05	0.640	± 0.08
	250	64.4	136.3	−466	132.16	1.54	143.7	71.64	0.716	± 0.09
	500	164.1	95.3	−462	93.03	1.08	281.5	80.04	0.800	± 0.04
AG	125	82.1	116.9	−475	91.83	1.07	228.1	80.29	0.802	± 0.06
	250	155.8	80.8	−423	52.22	0.61	442.6	88.79	0.888	± 0.07
	500	73.3	103.9	−469	40.08	0.47	457.3	91.4	0.914	± 0.04

The protective film formed by the inhibitors acts as an effective barrier, preventing contact between the CS surface and corrosive medium. At 500 ppm in 1.0 M HCl, the inhibition efficiency reaches 80.0% for G and 91.4% for AG, confirming AG’s effectiveness as a corrosion inhibitor for CS in HCl. The variation in inhibition efficiency between G and AG can be attributed to the difference in their alkyl group.

Table 4 shows the selected literature-reported inhibitors that possess similar functional groups—such as heterocyclic structures and atoms like nitrogen, sulfur, or oxygen. The comparison was carefully made using studies performed under closely matched experimental conditions, including acid concentration (1.0 M HCl), temperature (room temperature), and the type of metal substrate (CS), to maintain reliability and significance.

This comparative evaluation shows that the investigated inhibitors exhibit inhibition efficiencies that are on par with or exceed those of analogous compounds. These findings place our results in a broader context and emphasize the critical role of structural features in corrosion inhibition performance.

**Table 4:** Comparison of Inhibition efficiency of carbon steel in 1.0 M HCl.

compound	IE%	Reference
2-(2-hydroxy-5-nitrobenzylideneamino)phenol	80.0%	^33^
6-bromo-3- nitroso-2-phenylimidazol[1,2-a]pyridine	90.0%	^27^
2-hydrazino benzothiazole	73.0%	^34^
yrazole derivative (B	90.0%	^35^
imidazoline inhibitor	80.1%	^36^
3, 5-dimethyl-1 H-pyrazol-1-yl) (4-((4-chlorobenzy-lidene) amino) phenyl) methanone)	89.5%	^37^
3-methyl-4-amino-5-mercapto-1,2,4-triazole	80.0%	^38^
1-(benzo[*d*]thiazol-2-yl)−3-methylguanidine (AG)	91.4%	Our work

### Adsorption isotherm

Adsorption plays an essential role in the inhibition of metallic corrosion by organic molecules. It is widely accepted that the adsorption of inhibitors on the metal surface reduces the surface area available for electrode reactions, thereby hindering the corrosion process. Many researchers have employed the Langmuir adsorption isotherm to characterize inhibitors, assuming uniform adsorption and monolayer formation. To elucidate the interaction mechanisms, such as chemisorption and physisorption, of the inhibitor molecules (G and AG) on CS surface, various adsorption isotherms- including Langmuir, Temkin, Frumkin and Flory–Huggins- were applied to identify the most suitable model for describing the adsorption behavior of G and AG.

The surface coverage values θ, derived from Tafel polarization at varying concentrations of G and AG in 1 M HCl solution, were analyzed to identify the most appropriate adsorption isotherm for describing adsorption process. The adsorption of organic corrosion inhibitor at metal-solution interface involves a substitution mechanism, where organic molecules replace water molecules or other adsorbed species on the metallic surface. Within the studied concentration range, the experimental data showed the best correlation with the Langmuir adsorption isotherm, suggesting monolayer formation of uniform adsorption behavior^39^. The adsorption process may involve physical adsorption (physisorption), characterized by weak van der Waals forces, or chemical adsorption (chemisorption), which involves the formation of stronger covalent or coordinate bonds between the inhibitor molecules and the metal surface^32^.

Here, the Langmuir adsorption isotherm mono layer adsorption is presented in Eq. (3

3 $$\:\left(\frac{C}{\theta\:}+\right)=\frac{1}{K_{ads}}+C$$

Where C is inhibitors concentrations (mol/L), the surface coverage (θ) calculated from the PDP technique and K_ads_ is the equilibrium constant of the adsorption and calculated from the intercept of the straight line of the isotherm.

**Fig. 6:**
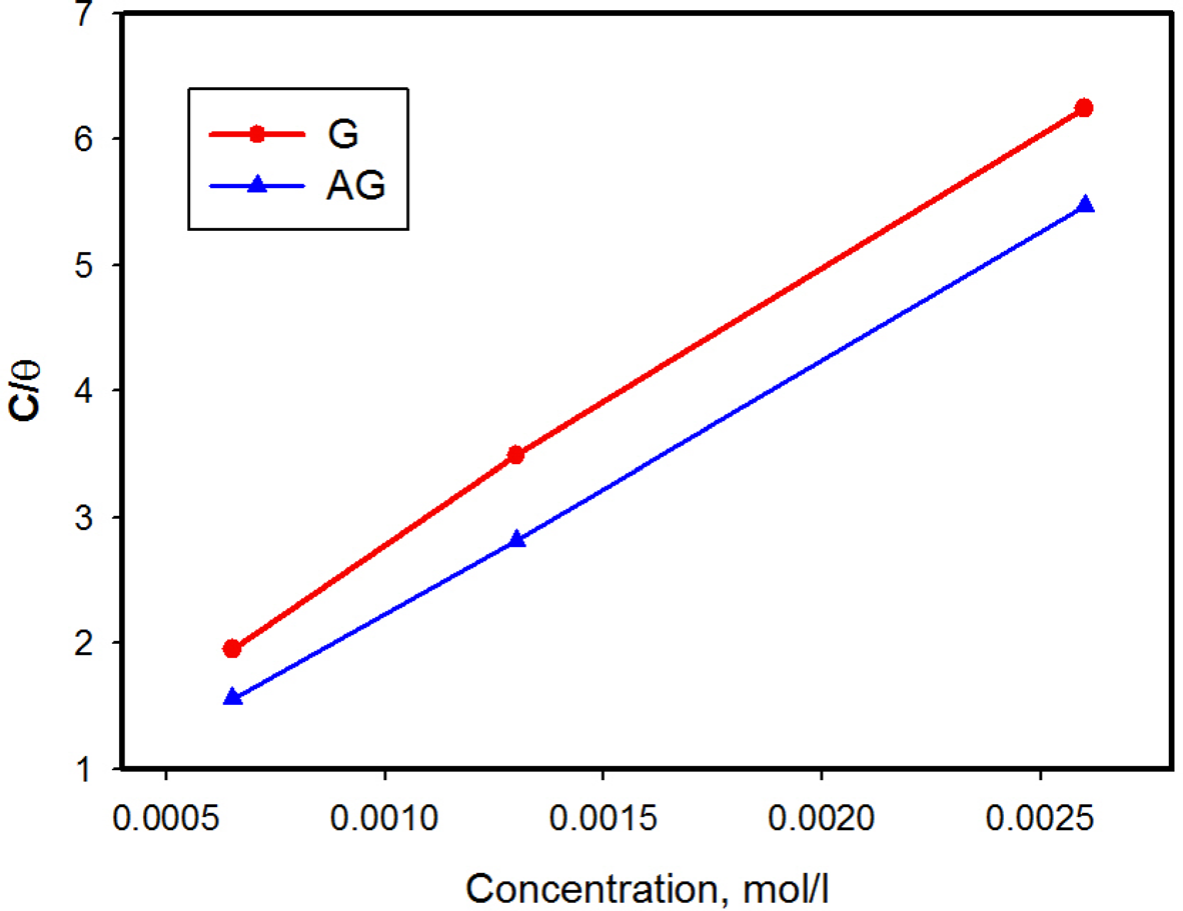
Langmuir adsorption isotherms for the adsorption of G and AG on carbon steel surface.

The degrees of the surface coverage for the different concentrations were evaluated from potentiodynamic polarization. The Langmuir isotherm was found to graphically fit the experimental data. The plot of *C/θ* vs. C as shown in Fig. 6 give a straight lines suggesting the adsorption of G, AG followed Langmuir isotherm, with *R* value of the order of 0.990 and 0.999 and the slop near the unity. From the intercepts of the straight lines with the *C*_inh_/*θ* axis, the values of *K*_ads_ were calculated. The K values describe the adherence force between the inhibitors and carbon steel surface^40^.

The standard free energy (∆ *G*_ads_) can be calculated by the following Eq^7^.

4 $$\Delta G_{o\:ads}=\:-RT\:{\rm ln}\:\left(\:55.5x\:K_{ads}\right)$$

Where 55.5 is the concentration of water in solution (mol/L), *R* is the universal gas constant 8.314 (J/mol K) and *T* is the absolute temperature.

The calculated values of ∆*G* are all negative and within the range − 34.3 and 35.3 kj mol^−1^. The negative values suggested that the inhibitor realize the spontaneous process.

The values of ∆G provide adsorption isotherm as physisorption, chemisorptions or both kind of adsorption. Values equal − 40 or more is taken as chemisorptions i.e. charge transfer or sharing from the inhibitors to the CS surface forming a kind of coordinate bond, while values equal to −20 kjmol^−1^or less is interpreted to suggest physisorption involve electrostatic interaction^41^, which reveals that the inhibitor molecules adsorb on the CS surface by both electrostatic (л electrons of aromatic rings) and charge transfer (S and –NH) process.

### Scanning electron microscopy (SEM&EDX)

**Fig. 7:**
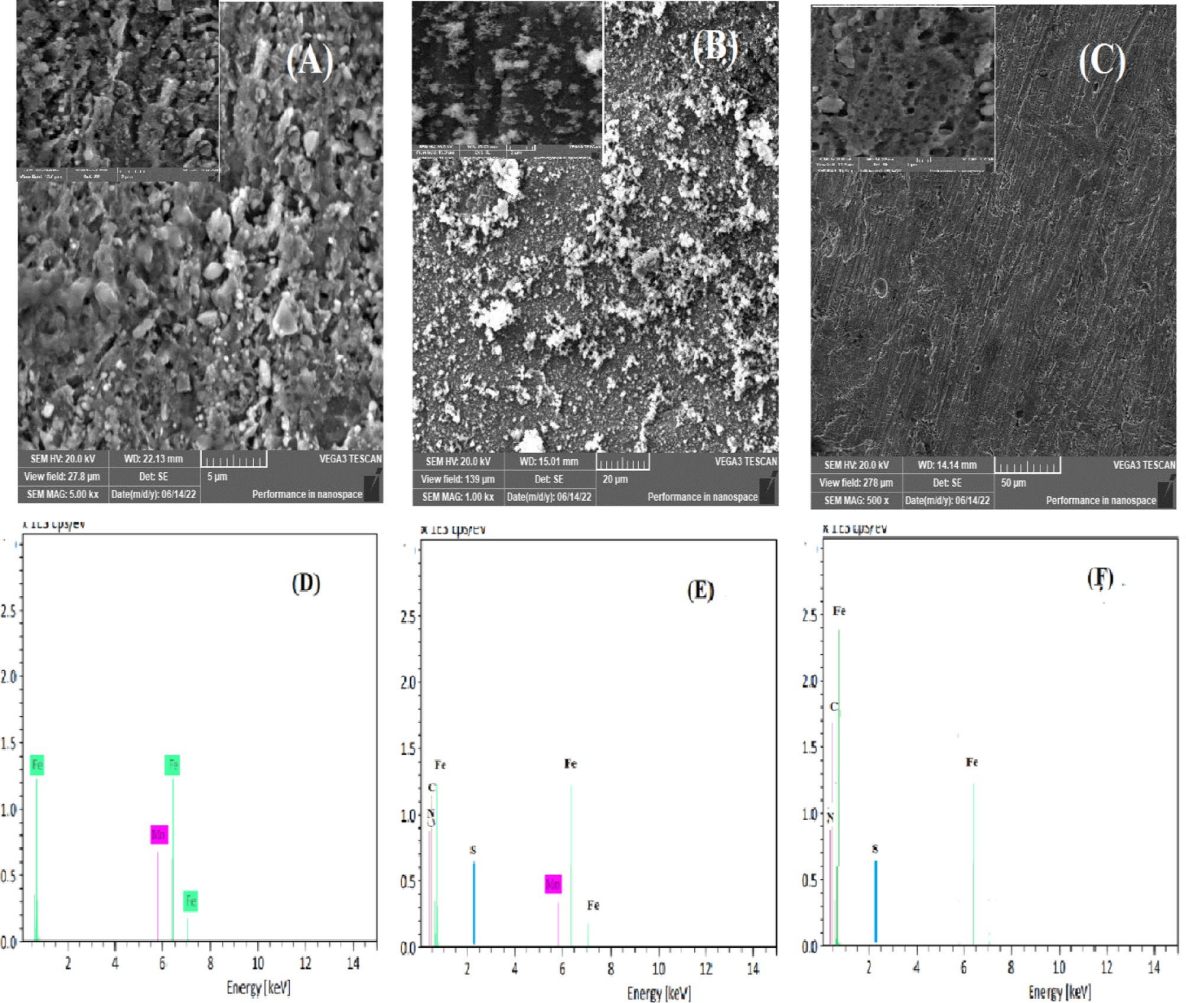
SEM images and EDAXspectra of surface morphologies of carbon steel in 1.0 M HCl in the presence and absence of different concentrations of inhibitors G and AG at room temperature.

SEM image were recorded in order to establish the interactions of the inhibitor on the CS surface in 1.0 M HCl. Figure 7 (A-C) shows the surface morphologies in three different specimens were immersed in 1.0 M HCl for 3 h without and with 500 ppm of G and AG, with inset images at different magnifications. Figure 7A shows the uninhibited specimen, heavily covered with corrosion products and exhibiting significant surface damage caused by the aggressive attack of chloride ions leading to a rough surface. On the other hand, Fig. 7B representing the sample treated with 500 ppm G, reveals the formation of flake-like crystallites, resulting in a smoother and denser layer with noticeably less surface damage. Figure 7C, depicting the surface treated with 500 ppm AG, demonstrates even greater protection, attributed to forming of a robust surface film offering higher corrosion resistance.

The corresponding EDX Spectra (Fig. 7D-F) highlight the elemental composition of carbon steel samples, showing peaks for Fe, Mn, and C along with oxygen. In Fig. 7D, the major element of the corrosion product is Fe with traces of Mn indicating severe corrosion in the uninhibited sample. In contrast, Fig. 7E and F, representing samples treated with 500 ppm of G and AG, respectively, show the presence of S and N, which coming from inhibitors.

These elements contribute to enhanced passivity and corrosion resistance of CS surface. The smoother, homogenous protective layer observed in Fig. 7F demonstrates that AG provides superior inhibition compared to G.

These findings corroborate the result from PDP, EIS analyses.

### Quantum calculations

**Fig. 8:**
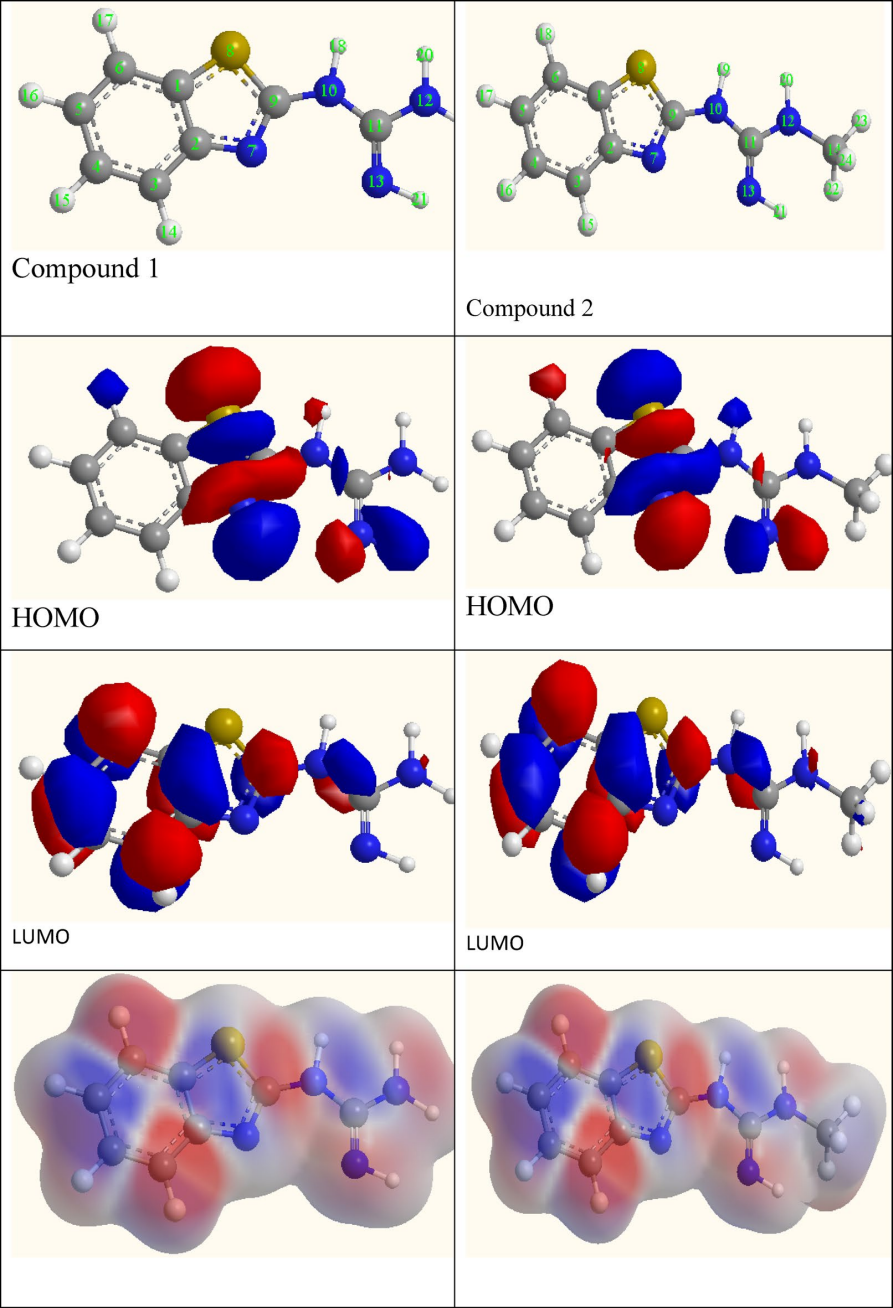
The highest occupied and lower unoccupied molecular orbitals (HOMO and LIMO) of the studied inhibitors (G and AG).

The lower and higher molecular orbital of investigated neutral and protonated inhibitors molecules are represented in Fig. 8. In general, higher E_HOMO_ and lower E_LUMO_ values are associated with enhanced adsorption capability.The value of *E*_HOMO_ and *E*_LUMO_ are detected for G and AG and found to be −7.806 eV and − 7.893eVfor the neural and − 8.120 eV and − 8.245 eV for the protonated, respectively. *E*_HOMO_ is attributed to the tendency of the molecule to donate its electron for the d orbital of the acceptor (carbon steel). While the ability of metal to donate back-donation described by *E*_LUMO_. Inhibitors that possess both donation and back donation ability classified as effective inhibitors. Thus, AG can be described as excellent inhibitor compared by G. The previous results are confirmed also by the calculated back-donation energy (*E*_b−d_), where AG < G^19^.

Key global descriptors (see Table 5) including ionization potential (*I*), electron affinity (*A*), energy gap (ΔE), electronegativity (*X*), global hardness (η), global softness (*S*), electrophilicity index (ω), and the number of electrons transferred (ΔN) were calculated to provide insights into the electronic reactivity and inhibition behavior. AG possess lower value of *E*_g_, *x* and *η* value than G, these data reflect it’s great probability of e’s transfer to the vacant d orbital of carbon steel. Thus, all the previous quantum descriptors declare that AG can easily interact with the metal surface to form resistive layer^42^.

The results revealed that the protonated forms exhibit lower energy gaps, higher electrophilicity indices, and greater dipole moments than their neutral counterparts. The previous results indicate the enhancement of the chemical reactivity and stronger adsorption potential in acidic media. Specifically, AG (protonated) demonstrated the lowest energy gap (6.50 eV), highest softness (0.3077 eV^−^¹), and electrophilicity index (3.84 eV), suggesting its superior capacity to interact with the steel surface through both electron donation and back-donation mechanisms^43^. The ΔN values, representing the number of electrons transferred to the metal surface, further supported this trend, with AG (protonated) showing a ΔN of 0.308, indicative of effective electron transfer interactions. Additionally, the elevated dipole moments of the protonated species point to increased polarity, enhancing solubility and orientation for surface adsorption. These findings align well with the experimental inhibition efficiencies and confirm the significant role of protonation in enhancing the molecular reactivity and adsorption characteristics of the inhibitors under acidic conditions.

**Table 5:** The energy gap (*E*_*g*_), electronegativity (*x*), hardness (*η*), and the electrophilicity index (ω) of inhibitors for G and AG (neutral (N) and protonated (P) form).

	E_HOMO_ (eV)	E_LUMO_ (eV)	ΔEg (eV)	Dipole (D)	X (eV)	η (eV)	Ϭ (eV^−1^)	E_b−d_ (eV)	ω (eV)	ΔN
AG(N)	−7.893	−0.871	7.022	3.48	4.382	3.511	0.2848	0.877	1.53	0.3728
AG(P)	−8.245	−1.745	6.500	5.65	4.995	3.2500	0.3077	0.813	2.08	0.3085
G(N)	−7.806	0.427	8.233	4.38	3.689	4.1165	0.2429	1.029	1.13	0.4021
G(P)	−8.120	−1.002	7.118	6.12	4.561	3.5600	0.2809	0.89	1.64	0.3427

It has been demonstrated that atoms with the most significant negative charges will more readily share electrons with the metal surface with which they interact. Additionally, these studies have shown that the reactivity of these atom locations grows as the absolute value of charge density rises. As a result, the active sites by which these inhibitors adsorb onto carbon steel are likely the atoms with a strong negative charge in G and AG (see Tables 6 and 7. The results convincingly demonstrate that the active sites centered on nitrogen and conjugated carbon atoms^24,44^.

**Table 6:** Mulliken atomic charge of G.

Atom	Charge	Atom	Charge	Atom	Charge	Atom	Charge
C(1)	−0.065	C(7)	−0.564	N(13)	−0.867	H(19)	0.089
C(2)	0.102	C(8)	0.690	H(14)	0.019	H(20)	0.096
C(3)	−0.164	C(9)	0.202	H(15)	0.020	H(21)	0.113
N(4)	−0.144	S(10)	0.261	H(16)	0.012		
C(5)	−0.134	N(11)	0.367	H(17)	0.013		
C(6)	−0.148	N(12)	0.023	H(18)	0.075		

**Table 7:** Mulliken atomic charge of AG.

Atom	Charge	Atom	Charge	Atom	Charge	Atom	Charge
C(1)	−0.065	N(7)	−0.566	N(13)	−0.872	H(19)	0.075
C(2)	0.102	S(8)	0.689	C(14)	−0.059	H(20)	0.087
C(3)	−0.167	C(9)	0.199	H(15)	0.019	H(21)	0.115
C(4)	−0.146	N(10)	0.259	H(16)	0.019	H(22)	0.030
C(5)	−0.135	C(11)	0.345	H(17)	0.020	H(23)	0.040
C(6)	−0.152	N(12)	0.119	H(18)	0.013	H(24)	0.030

### Inhibition mechanism

Understanding the corrosion mechanism of the CS is key to predict the adsorption behavior of inhibitors in hydrochloric acid solution^21^. In such an environment, the inhibitors may exist in both neutral and protonated states, depending on their functional groups. G and AG species interacts with the metal surface through different mechanisms:

Neutral inhibitor molecules typically adsorb onto the CS surface by donating lone pair electrons from electronegative atoms (such as nitrogen, oxygen, or sulfur) or through π-electrons from aromatic systems to the vacant d-orbitals of iron atoms. This results in the formation of coordinate covalent bonds, indicative of chemisorption. Moreover, the molecular planarity and conjugation enhance π–d orbital interactions, increasing surface coverage and improving inhibition efficiency.

Electrostatic interactions also occur between the positively charged protonated inhibitors and the negatively charged CS surface due to chloride ions from HCl can initially adsorb onto the metal surface, forming negatively charged localized areas. These sites favor the attraction of protonated inhibitor species via electrostatic forces.

The strong inhibition efficiency of AG is due to the synergistic effects of functional groups CH_3_, S, NH. CH_3_ group is an electron donating group increases the electron density on the NH and S groups, improving physisorption adsorption. While weaker than chemisorption, this interaction contributes to the formation of a protective layer that limits the access of aggressive ions like Cl⁻ to the surface.

Methyl group increases the bulking of the inhibitor molecule, enhancing surface coverage on CS surface, providing more effective barrier against corrosive agents. Methyl group contributes to a hydrophobic barrier that water penetration. It also enhances physical adsorption through van der Waals forces due to increase molecular size. Sulfur (S) exhibits strong adsorption affinity for metal surface through coordination, π- backbonding, and stable bond formation, creating a dense protective film. The NH group can form hydrogen bonds, and the lone pair of electrons on nitrogen can coordinate with the metal atoms forming chemical bonds, which stabilizing the inhibitor layer on the metal surface, and enhances adsorption through cooperative interaction with sulfur. Concurrently, neutral molecules can chemisorb onto other surface regions. The coexistence of both adsorption modes creates a synergistic effect, enhancing the overall inhibition performance.

## Conclusion

In conclusion, the synthesized inhibitors of G and AG were characterized using FT-IR, ^1^H and ^13^C NMR. Potentiodynamic polarization measurement revealed a significant reduction in corrosion current density with a slight shift in corrosion potential, indicating that the inhibitors adsorb onto the CS surface and block the reactive sites. The formation of a protective layer was confirmed by SEM/EDX analysis, and the theoretical parameters (e.g., low energy gap and high dipole moment) support the proposed inhibition mechanism.

Increasing inhibitor concentration reduces the surface area available for corrosive species leading to greater surface coverage by inhibitor molecules. The inhibitors reduce corrosion through a combined mechanism of chemisorptions and physisorption, with their adsorption on CS surface following the Langmuir isotherm. Polarization studies confirm that G and AG act as mixed type inhibitors, effectively protecting CS in 1.0 M HCl solutions. Among the two, AG demonstrates superior efficiency, achieving maximum protection at 500 ppm.

The newly synthesized inhibitors showed notable corrosion protection performance, with AG achieving an efficiency of up to 91.4%. The inhibition effectiveness is closely linked to the molecular structure of the compounds, as confirmed by quantum chemical parameters such as HOMO–LUMO energy levels, energy gap, chemical hardness, and dipole moment, which highlight the compounds’ ability to adsorb efficiently onto the steel surface.

Overall, these results not only introduce two promising corrosion inhibitors but also provide valuable insights that support the rational development of environmentally friendly inhibitors based on guanidine and benzothiazole frameworks.

## Supplementary Information

Below is the link to the electronic supplementary material.

Supplementary Material 1

## Data Availability

All data generated or analyzed during this study are included in this published article.
