# Supplementary material for: Electrochemical performance of a novel 1-(benzo[d]thiazol-2-yl)-3-methylguanidine as effective corrosion inhibitor for carbon steel in 1 M hydrochloric acid
Source: Sci Rep. 2025 Aug 2;15:28196. doi: 10.1038/s41598-025-12838-2 (PMC12316920; doi:10.1038/s41598-025-12838-2)
Supplement: Supplementary file 1 — Supplementary Material 1 [file 41598_2025_12838_MOESM1_ESM.doc]

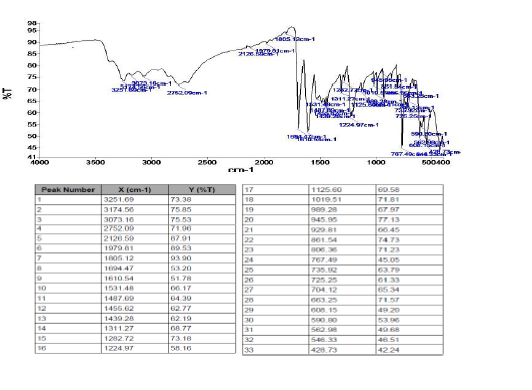


Supplementary S1: IR spectra of G compound


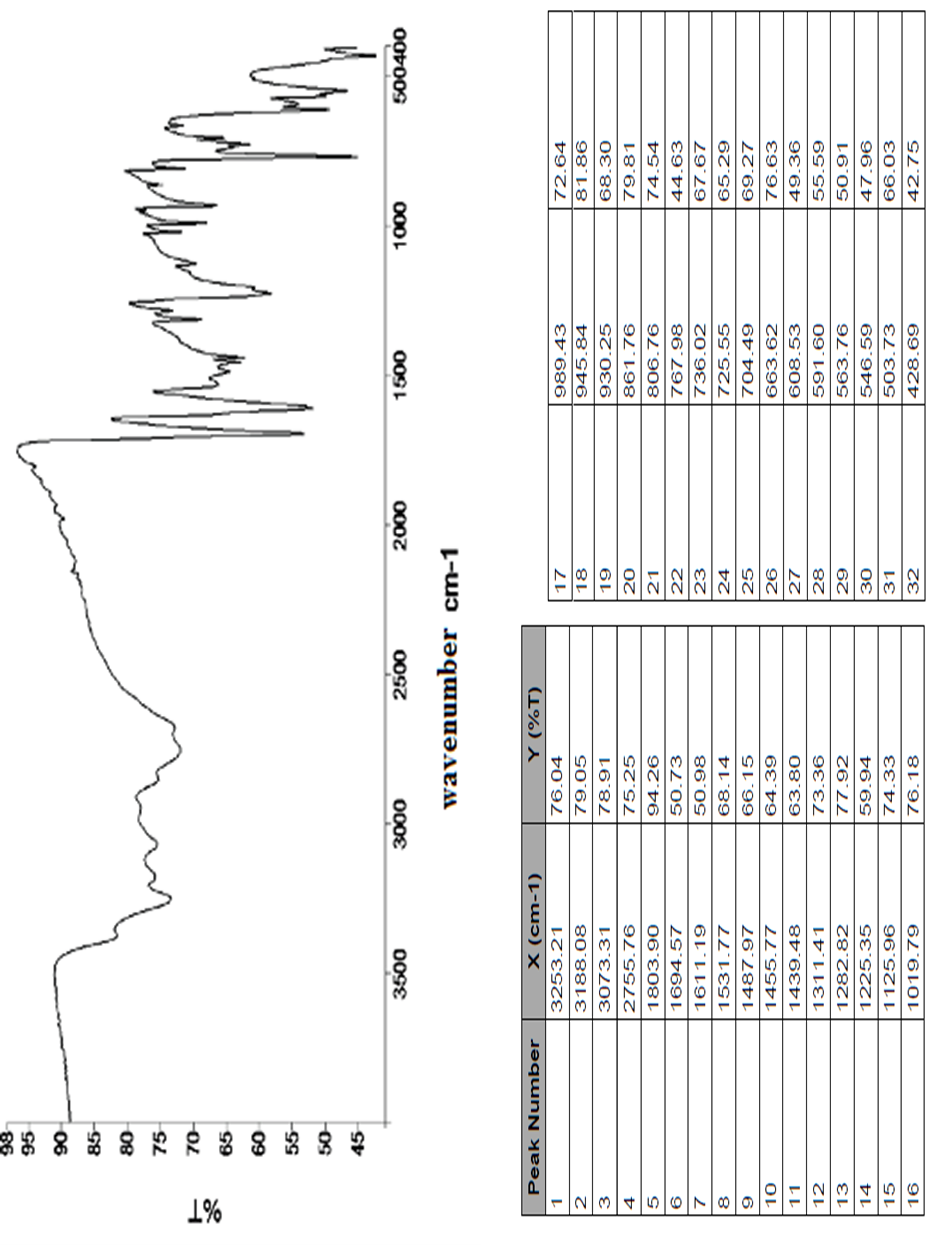


Supplementary S2: IR of compound (AG)


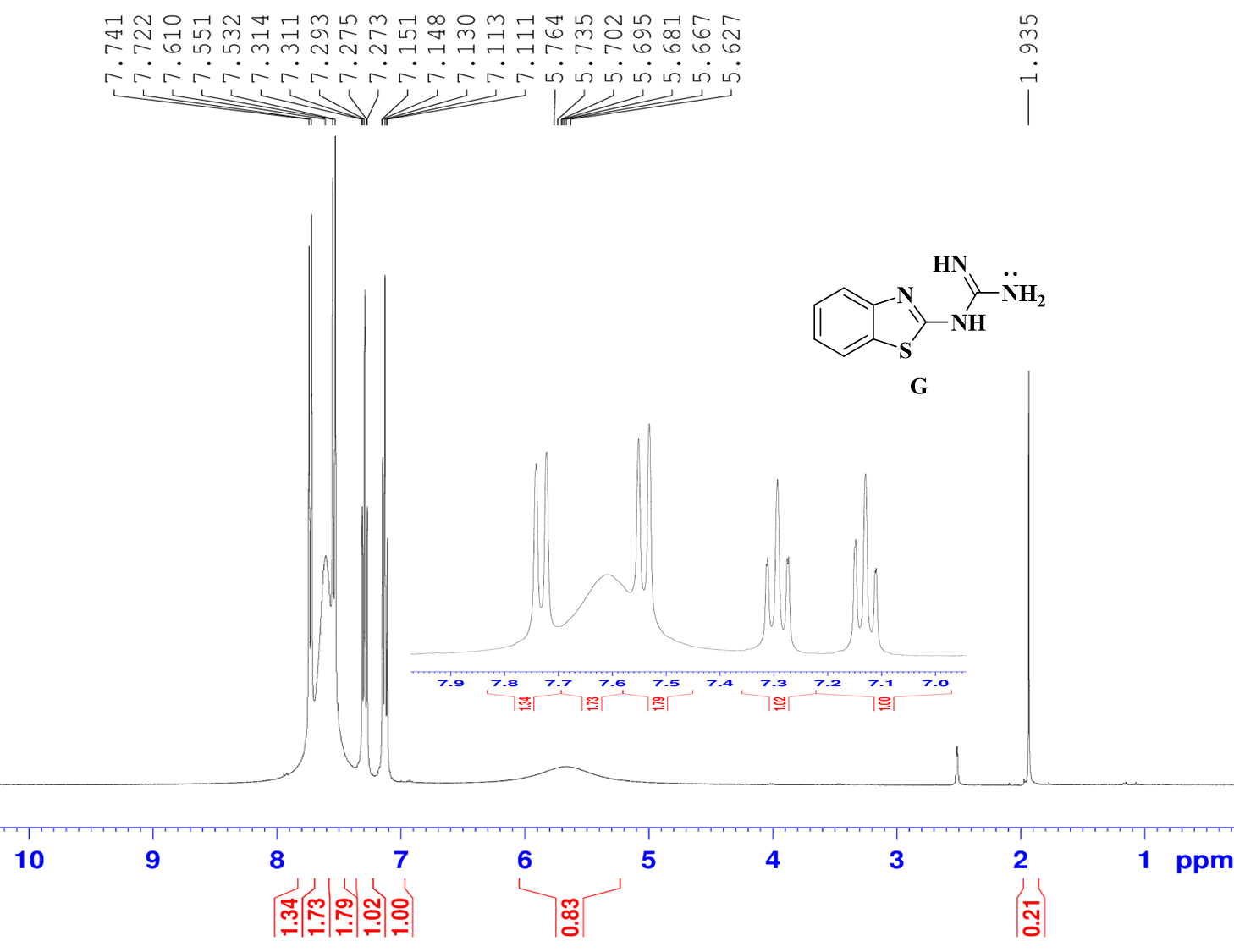


Supplementary S3:  ^1^H NMR of compound (G)


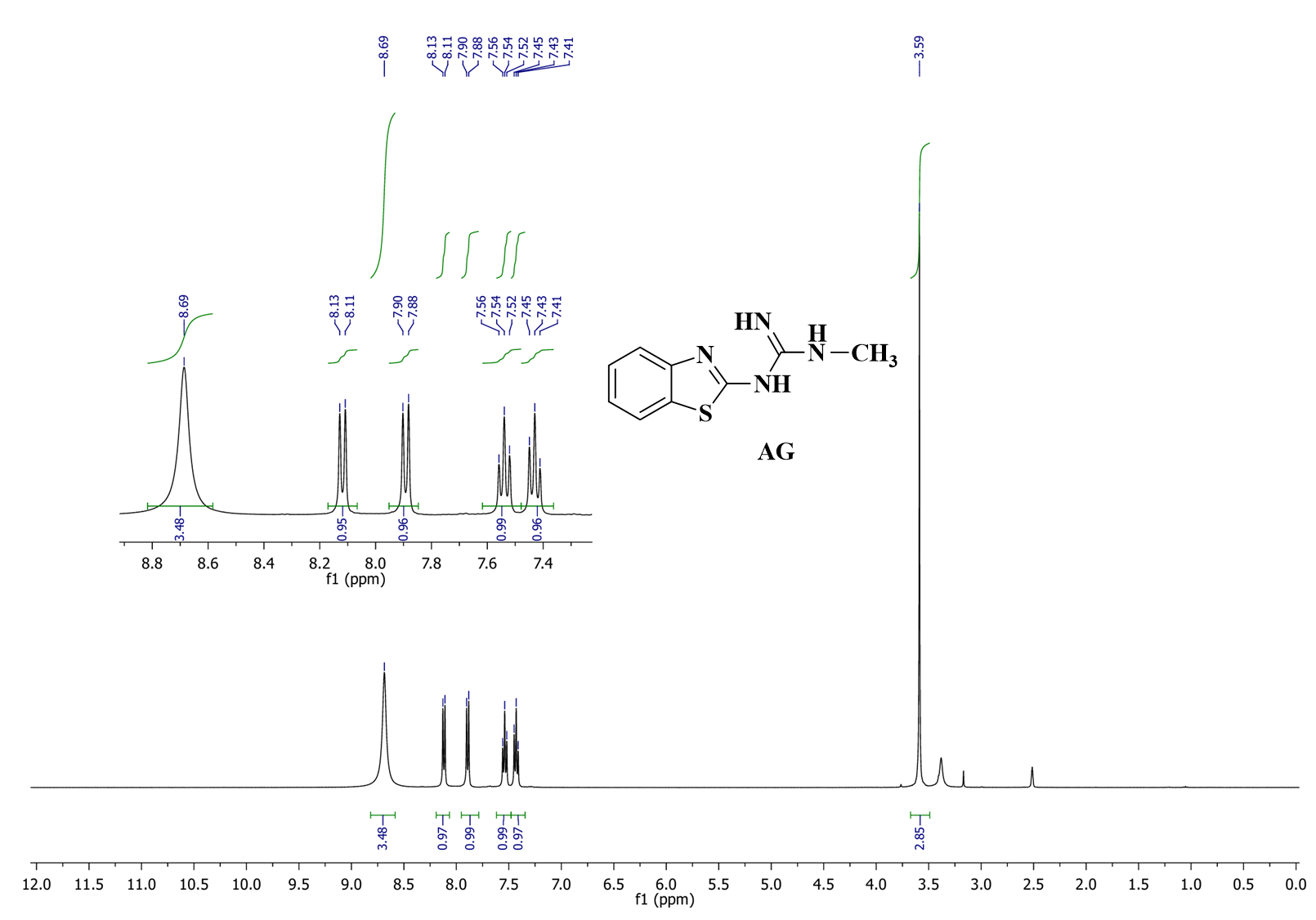


Supplementary S4 : ^1^H NMR of compound (AG)


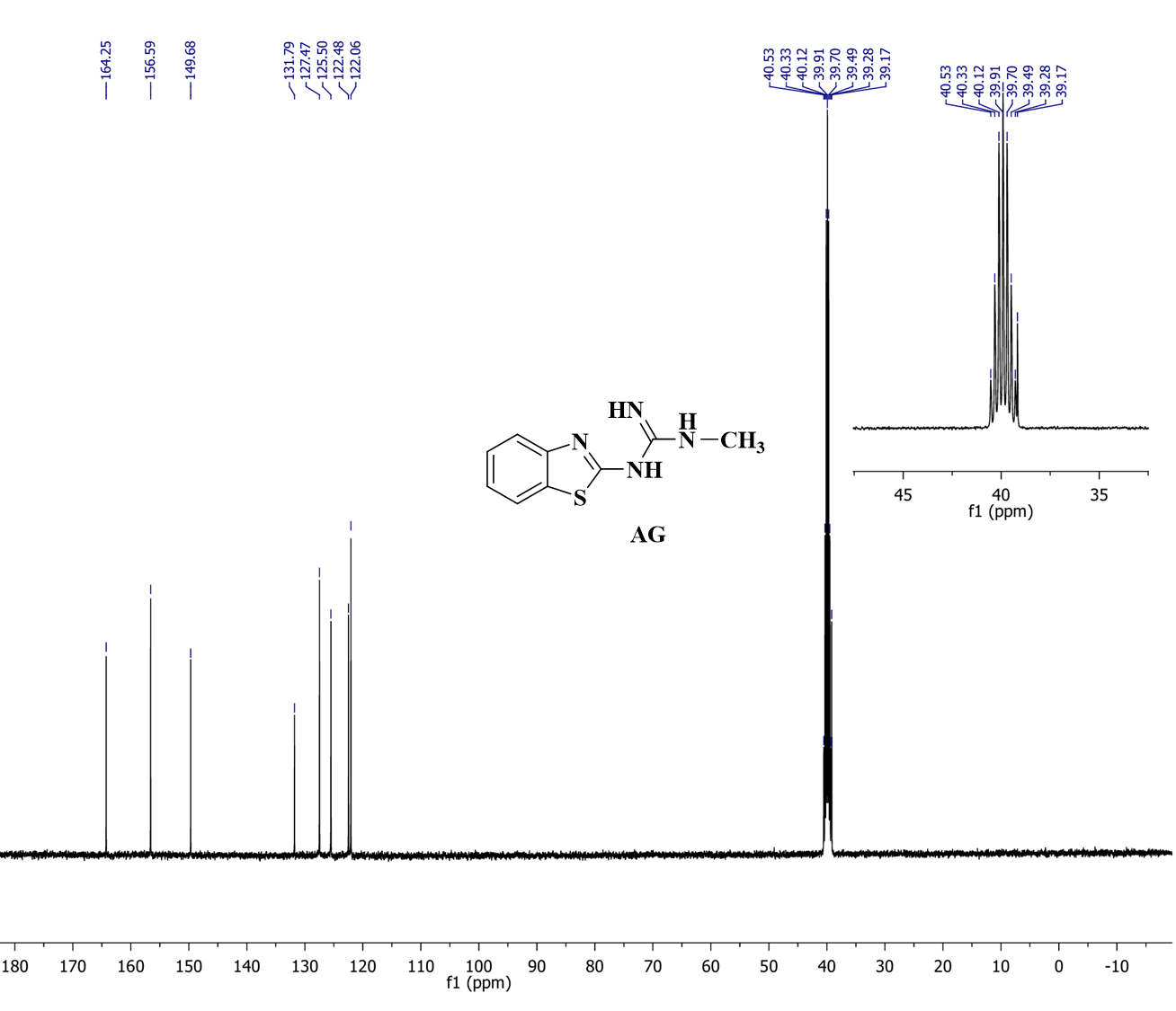


Supplementary S5: ^13^C NMR of compound (AG)
